# Supplementary material for: Sugar Starvation Disrupts Lipid Breakdown by Inducing Autophagy in Embryonic Axes of Lupin (Lupinus spp.) Germinating Seeds
Source: Int J Mol Sci. 2023 Jul 21;24(14):11773. doi: 10.3390/ijms241411773 (PMC10380618; doi:10.3390/ijms241411773)
Supplement: Supplementary file 1 [file ijms-24-11773-s001.zip › Figure S1.pdf]

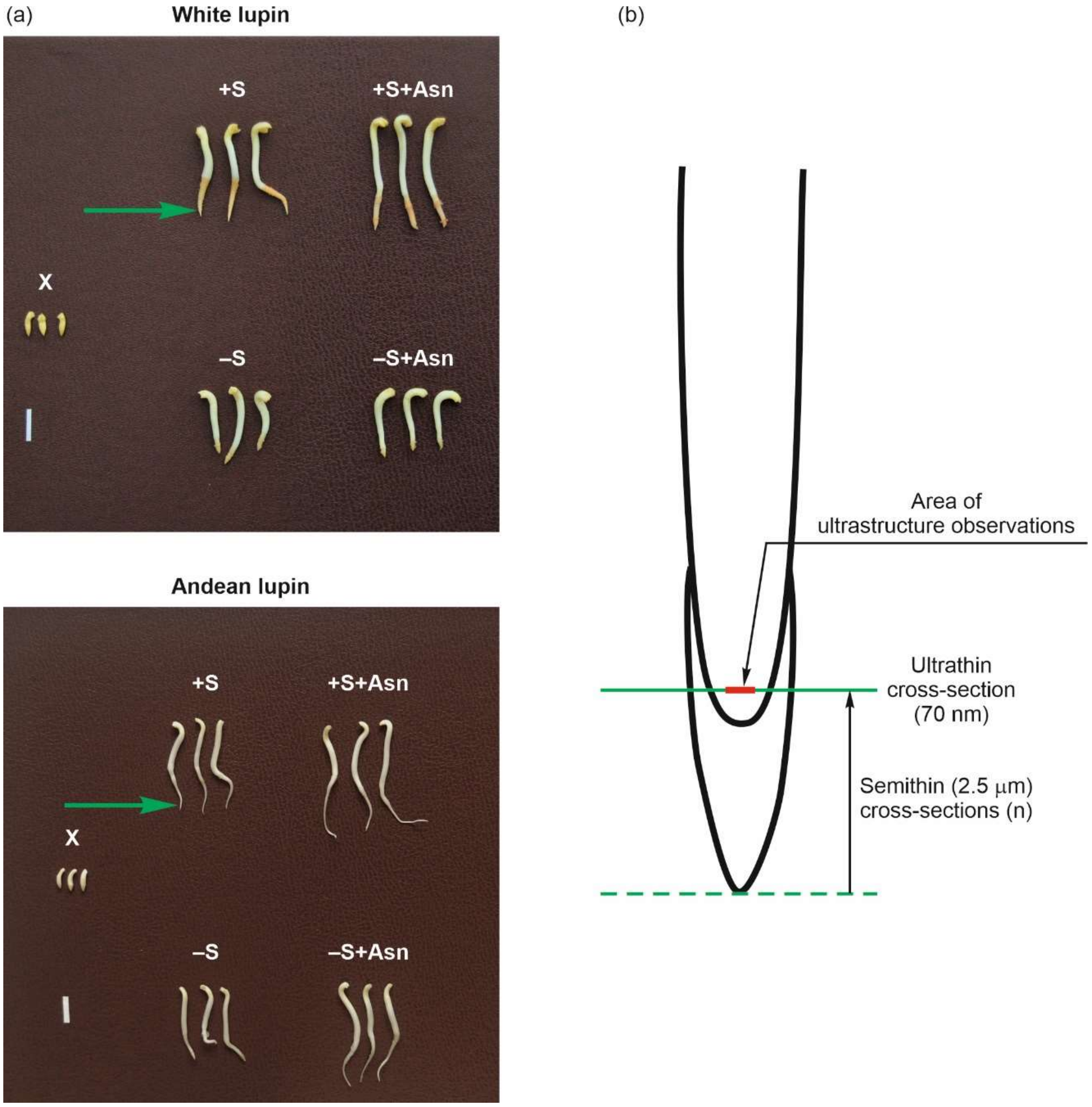

Figure S1. (a) Morphology of white and Andean lupin isolated embryo axes cultured *in vitro* for 96 h on a medium with (+S) and without (-S) 60 mM sucrose. Culture media were also enriched with 35 mM asparagine (+Asn). X - embryonic axes isolated from seeds after 24 h of imbibition; time 0 h of the *in vitro* culture. White vertical bar = 1cm. Green arrows indicate the region of ultrastructure observations. **(b)** Detailed localization of the root tip area selected for ultrastructure observations. The embedded in the epoxy resin 3-mm-long root tip of the cultured *in vitro* embryonic axis was consecutively cut from the tip of a root cap (green dashed line) into semithin sections (2.5 µm). Step by step, semithin cross-sections were analyzed under the light microscope. For the ultrastructural observations of the root meristematic zone cells under a transmission electron microscope (TEM), the level was chosen (green solid line) where in the central area of the cross-section small and closely packed cells were visible and this core was surrounded by cells of the root cap. These root cap cells were distinguishable from the central meristematic zone cell because they were bigger, loosely packed, contained large starch granules, were flacking off, and often mucus was visible around a section. Additionally, if the level was too shallow in the series of semithin sections, only cells of a root cap were visible under the light microscope, particularly columella cells, with clear and large granules of starch statoliths. When the level was too deep, the core of the root cross-section was composed of much bigger cells, they were not as closely packed as in the meristematic zone and they were not surrounded by flaking-off root cap cells. Depending on lupin species and a trophic variant of the *in vitro* culture, the number of removed semi-thin sections (n) was 125-250. Only the central area of such a selected level of root meristematic zone was analyzed under TEM (red insertion on the green solid line).
