# Supplementary material for: Sugar Starvation Disrupts Lipid Breakdown by Inducing Autophagy in Embryonic Axes of Lupin (Lupinus spp.) Germinating Seeds
Source: Int J Mol Sci. 2023 Jul 21;24(14):11773. doi: 10.3390/ijms241411773 (PMC10380618; doi:10.3390/ijms241411773)
Supplement: Supplementary file 1 [file ijms-24-11773-s001.zip › Table S5.pdf]

Table S5. Primer sequences used for qRT-PCR reactions. Actin was used as endogenous control. Primers were designed on NGS-derived sequences mapped to the reference genome.

Gene ID	Forward primer (5′-3′)	Reverse primer (5′-3′)	Amplicon length (bp)
		White lupin	
XM_019572931.1	gatgatggaaccttggcaacttatg	gccaaagcattcacttcatctg	122
XM_019559246.1	ggggactggtgcgaagaggtttc	caccaccctcagcttcggcgac	121
XM_019588469.1	ctatgtcacattttaggtacc	cttgagggtgttttggatgc	111
XM_019559290.1	caccagagagggtaactttgatc	cttggatgtgtgactttggg	122
XM_019562376.1	gaacacagaatcaaggtccg	gtgaagtctggacttccaaattc	119
XR_002105784.1	caaagcgacttctcggtgttac	gactcctgcatggcctccc	122
XM_019607520.1	cagaagagaagcaaagaggag	cctatcagaaacagatggtgg	121
XM_019570378.1	cgcattagagagaagtatccag	caaactgaccaacagtcaaatcag	124
XM_019585272.1 (actin)	ctcctgctatgtatgttgctatac	gagagcataaccttcatagatagg	128
		Andean lupin	
XM_019568374.1	gccaccaaccgcaattttcaac	cttccatcatcttttggaattg	125
XM_019591458.1	ggcaagggatgctatagggattac	gtgacccggtggaaaagactcg	130
XM_019588469.1	ctatgtcacattttaggcacc	cttgagggtttttcggatgc	111
XR_002105261.1	caccagagagggtaactttgatc	cctggatgtgtgacttcggg	122
XR_002105784.1	caaagcgacttctcggtgttac	gactcctgcatgacctccc	122
XM_019562376.1	gaacacagaatcaaggtccg	gtgaagtctggacttccaaattc	119
XM_019570378.1	ggcttcccgcattcgagag	caaactgaccaactgtcaaatccg	131
XM_019608702.1	ctagtgatttgggtgcaccttc	ggtcatagttttctttcactttgg	165
XM_019585272.1 (actin)	ctccggctatgtatgttgcc	gagagcataaccttcgtagatagg	128
