# Supplementary material for: Sugar Starvation Disrupts Lipid Breakdown by Inducing Autophagy in Embryonic Axes of Lupin (Lupinus spp.) Germinating Seeds
Source: Int J Mol Sci. 2023 Jul 21;24(14):11773. doi: 10.3390/ijms241411773 (PMC10380618; doi:10.3390/ijms241411773)
Supplement: Supplementary file 1 [file ijms-24-11773-s001.zip › Table S6.pdf]

Table S6. Summary of next-generation sequencing (NGS) quality.

**Sequencing 1**				
Library	Number of reads	Q30 (%)	Number of mapped reads	Percentage of mapped reads
White lupin, +S	45 587 123	96.06	42 471 145	93.16
White lupin, +S+Asn	75 948 271	96.68	70 922 341	93.38
White lupin, –S	15 467 709	96.26	14 230 241	92.00
White lupin, –S+Asn	20 310 261	96.77	18 797 080	92.55
Andean lupin, +S	39 345 348	96.95	36 164 291	91.95
Andean lupin, +S+Asn	65 753 503	96.66	60 522 222	92.04
Andean lupin, –S	34 578 149	97.17	31 275 002	90.45
Andean lupin, –S+Asn	42 588 111	96.77	38 724 774	90.92

**Sequencing 2**				
Library	Number of reads	Q30 (%)	Number of mapped reads	Percentage of mapped reads
White lupin, +S	46 031 705	94.54	43 180 370	94.44
White lupin, +S+Asn	39 473 364	94.67	37 252 632	94.55
White lupin, –S	44 164 948	94.73	41 437 811	94.01
White lupin, –S+Asn	35 916 260	94.85	34 092 432	94.95
Andean lupin, +S	43 696 551	94.70	40 804 505	93.59
Andean lupin, +S+Asn	44 140 263	94.52	41 699 581	94.57
Andean lupin, –S	44 834 214	94.95	41 865 586	93.44
Andean lupin, –S+Asn	46 946 551	94.78	43 476 282	92.71

**Sequencing 3**				
Library	Number of reads	Q30 (%)	Number of mapped reads	Percentage of mapped reads
White lupin, +S	72 881 182	93.92	68 751 715	94.35
White lupin, +S+Asn	69 088 233	93.49	65 420 544	94.70
White lupin, –S	62 811 687	93.67	58 951 293	93.88
White lupin, –S+Asn	71 554 132	93.91	67 052 522	93.73
Andean lupin, +S	65 278 260	93.57	61 087 355	93.00
Andean lupin, +S+Asn	68 275 501	93.81	64 287 264	94.20
Andean lupin, –S	66 363 521	93.77	62 707 272	94.50
Andean lupin, –S+Asn	61 970 297	93.50	57 762 638	93.27

Q30 (%), reads quality according to the percentage of bases having a base quality value greater or equal to 30, i.e. less than 1 error in 1000 bases.

+S, embryonic axes cultured 96 h *in vitro* on medium with 60 mM sucrose
–S, embryonic axes cultured 96 h *in vitro* on medium without sucrose
+Asn, embryonic axes cultured 96 h *in vitro* on +S or –S media enriched with 35 mM asparagine
